# Cognitive Impairment in Non-critical, Mild-to-Moderate COVID-19 Survivors

**DOI:** 10.3389/fpsyg.2022.770459

**Published:** 2022-02-17

**Authors:** Ashley M. Henneghan, Kimberly A. Lewis, Eliana Gill, Shelli R. Kesler

**Affiliations:** ^1^School of Nursing, The University of Texas at Austin, Austin, TX, United States; ^2^Department of Oncology, Dell Medical School, The University of Texas at Austin, Austin, TX, United States; ^3^Ascension Seton Medical Center, Austin, TX, United States; ^4^Department of Physiological Nursing, University of California, San Francisco, San Francisco, CA, United States; ^5^Department of Diagnostic Medicine, Dell Medical School, The University of Texas at Austin, Austin, TX, United States

**Keywords:** cognition, COVID-19, executive function, anxiety, psychosocial

## Abstract

**Importance:**

Previous studies of post-acute COVID-19 syndrome have focused on critical cases with severe disease. However, most cases are mild to moderate in disease severity.

**Objective:**

We aimed to examine cognitive outcomes in cases of non-critical, mild-to-moderate COVID-19. Design, Setting, and Participants: In this cross-sectional study, we enrolled 72 adults aged 22 to 65 years in Central Texas who had non-critical, mild-to-moderate COVID-19 infection between 13 January 2021 and 20 April 2021.

**Main Outcomes and Measures:**

We remotely administered cognitive-behavioral testing to determine the frequency of cognitive impairment and examine demographic, clinical, and psychosocial contributors to impairment.

**Results:**

The frequency of objective cognitive impairment was 40%. The largest number of participants (24%) showed impairment on a measure of executive functioning. Attention and processing speed was more impaired in males (*OR* = 1.5, 95%CI = 0.23–2.9). Males endorsed lower adherence to social distancing guidelines (*U* = 590, *p* = 0.01), which was in turn associated with cognitive impairment across participants (*r* = −0.30, *p* = 0.01). Younger age was correlated with impairment (*r* = −0.26, *p* = 0.03) but was also associated with racial/ethnic minority status (*r* = −0.31, *p* = 0.01) and increased psychological symptoms (*p* < 0.04). Greater number of COVID-19 symptoms was correlated with lower subjective cognitive function (*r* = −0.38, *p* = 0.001) as well as psychosocial function (*r* > 0.24, *p* < 0.05). Moderate COVID-19 severity was associated with attention/processing speed impairment (*r* = 0.27, *p* = 0.03), increased pain (*r* = 0.31, *p* = 0.01), and higher number of COVID-19 symptoms (*r* = 0.32, *p* = 0.01).

**Conclusion and Relevance:**

Mild or moderate COVID-19 infection may be associated with cognitive impairments, especially in the domain of executive functioning. A subgroup of younger individuals may be more vulnerable to cognitive and psychosocial effects of COVID-19.

**Highlights:**

Question: How frequent is cognitive impairment among non-critical, mild-to-moderate COVID-19 survivors?

**Findings:**

In this cross-sectional study of 72 adults, 40% demonstrated cognitive impairment, particularly in executive function.

**Meaning:**

Neurologic sequelae, such as cognitive impairment, may be common following COVID-19 infection.

## Introduction

Although COVID-19 is an acute respiratory syndrome, it has direct and indirect effects on neurobiology that can result in cognitive impairment as well as other neurologic symptoms (Moro et al., 2020). Cognitive impairment can significantly decrease quality of life, interfering with educational, occupational, and psychosocial functioning as well as independence and adaptive functioning (Abrahamson et al., 2012; Mayo et al., 2020). Miskowiak et al. (2021) observed that cognitive impairments, especially in executive function, were strongly correlated with decreased quality of life as well as greater psychological distress in patients with COVID-19 (Miskowiak et al., 2021).

Research regarding the effects of COVID-19 on cognitive function is still very nascent but studies utilizing objective cognitive testing indicate deficits across several cognitive domains including attention, processing speed, visual and verbal memory recall, executive function, and psychomotor coordination (Zhou et al., 2020; Ferrucci et al., 2021; Hampshire et al., 2021; Jaywant et al., 2021; Mazza et al., 2021; Miskowiak et al., 2021; Ramani et al., 2021). Several studies have also indicated significant cognitive impairment *via* qualitative clinical observation of cognitive function (Helms et al., 2020; Pinna et al., 2020; Varatharaj et al., 2020). The incidence of cognitive impairment observed across studies ranged from 28–81% of patients. The broad range likely reflects differences in patient samples, cognitive measurements, and definition of cognitive impairment.

Post-acute COVID syndrome is not limited to severe cases but is also very prevalent among those with mild-to-moderate disease (Amenta et al., 2020; Rubin, 2020; Moreno-Perez et al., 2021). A recent study demonstrated significant cognitive impairment among non-critical cases, although the effect sizes were small (Hampshire et al., 2021). However, most studies conducted to date have examined patients with severe disease who were hospitalized for COVID-19. Critical patients tend to receive more intensive treatments including mechanical ventilation, and ICU admission is associated with significant cognitive deficits, especially in those with respiratory distress (Honarmand et al., 2020). Certain mechanisms underlying cognitive deficits may depend on disease severity. For example, hypoxia may play a larger role in severe cases and studies have indicated a relationship between respiratory symptoms and COVID-related cognitive impairment (Hampshire et al., 2021; Miskowiak et al., 2021). Further, severe COVID-19 cases may have higher rates of comorbidities, such as obesity and diabetes, that are known to independently contribute to cognitive decline (Dye et al., 2017; Xue et al., 2019).

Considering that most COVID-19 cases are mild to moderate, determining potential cognitive sequelae and demographic/clinical correlates is critical. The current study examined both objective and subjective cognitive function, emotional distress, fatigue, sleep disturbance, social functioning, demographic and clinical variables, and self-reported social distancing behaviors in adults with a history of non-critical, mild-to-moderate COVID-19. We determined the frequency of cognitive impairment and examined potential demographic, clinical, and psychosocial contributors to impairment.

## Materials and Methods

### Participants

A local hospital in Central Texas identified positive COVID-19 cases that were evaluated between March 2020 and July 2020. The study was also advertised *via* social media. Adults (ages 21–75) who self-reported testing positive for COVID-19 and not being hospitalized for COVID-19-related symptoms/treatment and were willing and able to complete remote data collection were included. Those with a pre-COVID diagnosis of neurological or psychiatric disorders or who could not speak/read English or Spanish were excluded. The study was approved by the University of Texas at Austin Institutional Review Board.

### Demographics, Medical, and COVID-19 Clinical History

We used instruments suggested by the National Institutes of Health to facilitate COVID-19-related research as included in the PhenX Toolkit (Pan et al., 2012). We used the OSUMC Impact Questionnaire to measure demographics, UPenn Patient Health-General Health Questionnaire to measure health history, and the COVID-19 Experiences (COVEX) questionnaire to measure COVID specific diagnosis, symptoms, and behaviors (NIH Repository of COVID-19 Research Tools). The COVEX questionnaire defines illness severity as mild (“dry cough, headache, nausea/diarrhea, aches and pains, low-grade fever, no need to see a doctor or hospitalization”), moderate [“coughing, high fever (above 100.0^o^ Fahrenheit or 37.8^o^ Celsius), chills, feeling that you cannot get out of bed, shortness of breath],” severe (“breathlessness, complications leading to pneumonia”), and critical (“respiratory failure, septic shock, and/or organ dysfunction or failure”).

### Cognitive Functioning

We administered BrainCheck, an FDA approved, web-based battery of standardized neuropsychological tests that has been shown to have high sensitivity and specificity for mild or greater cognitive impairment (Ye et al., 2020). BrainCheck requires approximately 15 min to complete and is available in both English and Spanish. BrainCheck includes the Trail Making Test for executive function (cognitive flexibility), the Digit Symbol Substitution Test for attention and processing speed, the Stroop Test for executive function (response inhibition), and the List Learning Test for immediate and delayed verbal memory (Groppell et al., 2019). BrainCheck scores have a normative mean of 100 with a standard deviation of 15. We defined objective cognitive impairment as a score that was 1.5 standard deviations or more below the normative mean on one or more of the BrainCheck tests. This is midway between prior studies which tended to use 1.0 or 2.0 standard deviations to define impairment (Jaywant et al., 2021; Miskowiak et al., 2021).

The Patient Reported Outcome Measures Information System v2.0 Cognitive Function Short Form 8a (PROMIS Cognitive) was administered online *via* REDCap Survey (Vanderbilt, TN) to assess subjective cognitive function (Jensen et al., 2017). Scores for this measure have a normative mean of 50 with a standard deviation of 10. Subjective cognitive impairment was defined as a PROMIS Cognitive score that was 1.5 standard deviations or more below the normative mean.

### Psychosocial Symptoms

The PROMIS 57 (Cella et al., 2010) was administered to evaluate symptoms of depression, fatigue, anxiety, sleep disturbance and pain interference, physical functioning, and social role performance. Raw scores were used in analyses. The Perceived Stress Scale was used to measure stress (Cohen et al., 1983). Total scores for this 10-item scale can range from 0 to 40, with higher scores indicating more perceived stress. All testing and questionnaires were offered in English or Spanish. Given the pandemic-related shutdown, this study was conducted entirely remotely, *via* videoconference.

### Data Analyses

Descriptive statistics were used to describe demographic and clinical variables. We calculated the frequencies of objective and subjective cognitive impairment. We examined potential contributors to impairment *via* Pearson/Spearman correlation, chi-square, and Mann–Whitney U-test, as appropriate. Contributors evaluated included age (years), sex (1 = female, 0 = male), education (years), racial/ethnic minority status (1 = yes, 0 = no), number of COVID-19 symptoms, self-rated COVID severity (0 = mild, 1 = moderate), time since COVID-19 diagnosis (months), self-rated compliance with social distancing guidelines (1 to 5 points with 5 being highest compliance), and psychosocial functioning (anxiety, depression, perceived stress, sleep disruption, pain, fatigue, social function). Alpha level was set at *p* < 0.05. Analyses were conducted in the R Statistical Package v4.1.0 (Vienna, Austria).

## Results

We enrolled 105 adults between 13 January 2021 and 20 April 2021. For this analysis, we focused on the 79 participants who self-reported testing positive for COVID-19 and having mild-to-moderate disease severity. After further examination of participant’s self-report data, seven were excluded for endorsing days spent in hospital for COVID-19 treatment resulting in a final sample size of 72. Participants were on average 3.8 months post-diagnosis (+/− 3.1 months), age from 22 to 65 years (mean = 36 +/− 12 years), 74% were female and 42% reported racial/ethnic minority status. Participants tended to be highly educated (mean = 17 years of education +/− 2 years) and 100% spoke English and elected to complete tests and questionnaires in English (Table 1).

**Table 1:** Demographic and clinical characteristics.

	*n*	%	Mean	Standard deviation	Minimum	Maximum
Age			36.28	12.0	22	65
Education			16.89	2.16	12	24
English speaking	72	100%				
Months since COVID diagnosis			3.8	3.2	0	10
Minority Status	30	42%				
Female Sex	53	74%				
Number of COVID-related symptoms during infection			7.36	3.5	1	16
COVID Severity Mild Moderate	45 27	63% 37%				

### Cognitive Impairment

Results indicated that 40% of participants demonstrated objective cognitive impairment. The largest number of participants showed impairment on the Stroop test (24%), a measure of executive functioning (Table 2). Fifteen percent of participants endorsed subjective cognitive impairment.

**Table 2:** Cognitive-behavioral outcomes (*N* = 72).

	Mean	Standard Deviation	Minimum	Maximum	Impaired	
					N	%
**BrainCheck^a^**						
Trails A	99	17	55	126	9	13%
Trails B	102	15	42	132	3	4%
Digit Symbol	98	19	37	137	12	17%
Stroop	91	21	35	136	17	24%
Immediate Recall	101	20	2	117	8	11%
Delayed Recall	98	25	0	117	11	15%

**PROMIS Cognitive^a^**	45	10	24	64	11	15%

**PROMIS 57**						
Depression^b^	14	7	8	32		
Anxiety^b^	18	8	8	37		
Sleep Disruption^b^	19	7	8	38		
Fatigue^b^	20	9	8	40		
Pain^b^	12	7	7	40		
Physical Functioning^a^	36	6	12	40		
Social Role^a^	30	8	8	40		

Perceived Stress Scale^b^	17	8	1	34		

PROMIS, Patient Reported Outcome Measures Information System. BrainCheck tests have a normative mean of 100 and a standard deviation of 15. The PROMIS Cognitive test has a normative mean of 50 and a standard deviation of 10. PROMIS 57 and Perceived Stress Scale scores are presented as raw scores.

a Lower scores = lower function.

b Lower scores = fewer symptoms.

### Contributors to Cognitive Impairment

There was no difference in objective cognitive impairment between males and females but Digit Symbol, a measure of attention and processing speed, was more impaired in males (*X*^2^ = 5.86, *p* = 0.02, Table 3). There were no gender differences in age, education, minority status, COVID-19 symptoms/severity, time since diagnosis or psychosocial function. However, males rated themselves as being less compliant with social distancing guidelines compared to females (*U* = 590, *p* = 0.01) and there was an association between lower compliance and higher overall cognitive impairment across participants (*r* = −0.30, *p* = 0.01). Given these findings, we conducted a post-hoc analysis to determine if gender mediated the relationship between social distancing compliance and objective cognitive impairment. The lavaan library (Rosseel, 2012) in the R Statistical Package was used with diagonally weighted least squares estimation, delta method standard errors and bias corrected bootstrap confidence intervals (1,000 replications). The unstandardized indirect effect was −0.01 (95% confidence interval: −0.36–0.37, *p* = 0.96).

**Table 3:** Sex and objective cognitive impairment.

	Male (*n* = 16)	Female (*n* = 53)	*X* ^2^	value of *p*
Trails A impairment	1 (6%)	8 (15%)	0.89	0.35
Trails B impairment	0 (0%)	3 (6%)	0.99	0.32
Digit Symbol impairment	6 (38%)	6 (11%)	5.9	0.02
Stroop impairment	5 (31%)	11 (21%)	0.76	0.38
Immediate Recall impairment	2 (13%)	5 (9%)	0.13	0.72
Delayed Recall impairment	2 (13%)	9 (17%)	0.18	0.67
Any objective cognitive impairment	8 (50%)	21 (40%)	0.54	0.46

Data are shown as *N* (percentage).

There were no significant differences in frequency of objective cognitive impairment between those who identified as a racial/ethnic minority and those who did not (*X*^2^ = 2.6, *p* = 0.11). Surprisingly, younger age was correlated with objective cognitive impairment (*r* = −0.26, *p* = 0.03). However, younger participants had higher perceived stress (*r* = −0.32, *p* = 0.01), anxiety (*r* = −0.24, *p* = 0.04), and depressive symptoms (*r* = −0.26, *p* = 0.03). Additionally, younger age was associated with racial/ethnic minority status (*r* = −0.31, *p* = 0.01). Given these results, we conducted a post-hoc logistic regression which indicated that minority status and psychological distress reduced the effect of age on cognitive impairment (OR = 0.951, 95% CI = 0.90–0.99, *p* = 0.05). Minority status and psychological distress were not significant in the model singularly or as interaction terms with age. Although not significant, minority status had the largest effect size of any predictors (OR = 1.6, 95% CI = 0.56–4.5, *p* = 0.39). There were no significant relationships between age and social distancing compliance, COVID-19 symptoms/severity, or time since diagnosis. There were also no significant relationships between these variables and minority status.

**Figure 1:**
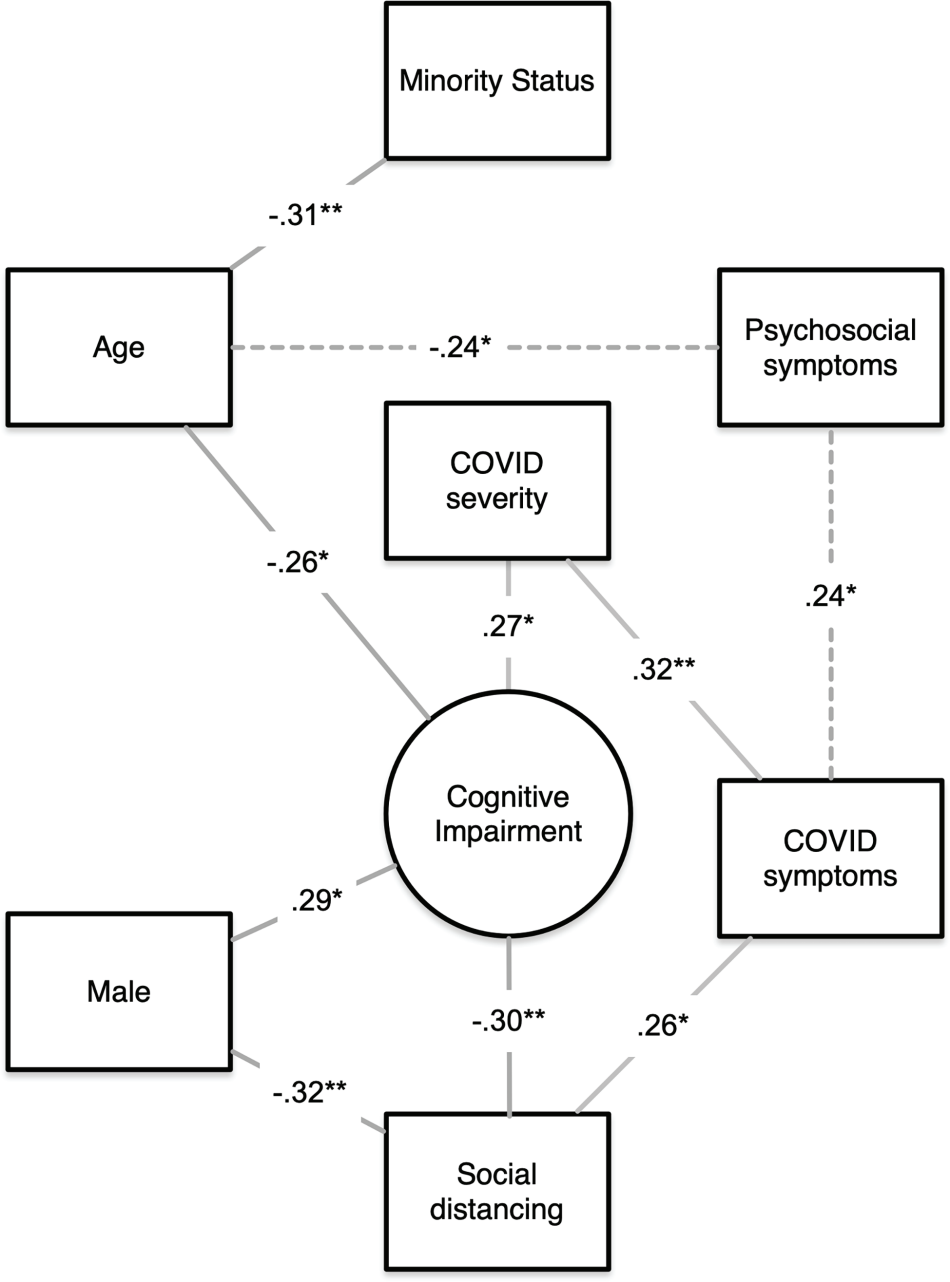
Summary of contributors to objective cognitive impairment. A visual summary of our findings showing the significant correlations among age, psychosocial symptoms, minority status, sex, number of COVID symptoms, COVID severity, social distancing, and objective cognitive impairment. Lines indicate that a significant correlation was noted between the two variables. Lines are labeled with the correlation coefficient, and the asterisk indicates the significance level (^*^p < 0.05, ^**^p < 0.01). Paths representing multiple correlations (dashed lines) are labeled with the minimum correlation coefficient only. Chi-square data for sex effects are expressed as correlation coefficients for consistency.

Anxiety, depressive symptoms, fatigue, and sleep disturbance were not associated with objective cognitive impairment but were related to subjective cognitive impairment (*p* < 0.001). Greater number of COVID-19 symptoms was correlated with lower subjective cognitive function (*r* = −0.38, *p* = 0.001) and social function (*r* = −0.28, *p* = 0.02) as well as higher anxiety (*r* = 0.32, *p* = 0.01), fatigue (*r* = 0.24, *p* = 0.05), and sleep disturbance (*r* = 0.26, *p* = 0.03). Moderate COVID-19 severity was associated with attention/processing speed impairment (*r* = 0.27, *p* = 0.03), higher number of COVID-19 symptoms (*r* = 0.32, *p* = 0.01), and increased pain (*r* = 0.31, *p* = 0.01). Education and time since diagnosis were not associated with any outcomes. See Figure 1 for a summary of contributors to cognitive impairment.

## Discussion

Few studies have examined the effects of COVID-19 on cognitive function but suggest that cognitive impairment is as high as 81% in severe cases requiring hospitalization. However, most COVID-19 cases are mild to moderate and even less is known regarding cognitive outcomes in these patients. We demonstrated cognitive impairment in 40% of non-critical, mild-to-moderate severity COVID-19 survivors. It is difficult to compare our results with prior findings given that previous studies have focused on severe cases (Helms et al., 2020; Ferrucci et al., 2021; Jaywant et al., 2021; Mazza et al., 2021; Miskowiak et al., 2021; Raman et al., 2021; Ramani et al., 2021). The median percentage impaired reported among these studies was 61%, and therefore, our results suggest that incidence of cognitive impairment is lower in mild-to-moderate cases.

Hampshire et al. conducted the largest study to date including 84,285 total COVID-19 cases. Most participants were approximately 1–3 months post-illness and completed online objective cognitive testing with one overlapping test, making this study the most similar to ours to date. Their findings indicated the worst cognitive impairment among those with the most severe respiratory symptoms, but they also demonstrated significant cognitive impairment among individuals who were not hospitalized and reported no respiratory symptoms (Hampshire et al., 2021). However, it is unclear what definition of cognitive impairment was used or what percentage of participants this involved.

Few if any other studies have utilized standardized neuropsychological testing in non-critical cases. One study involving 18 mild–moderate cases who were approximately 3 months post-infection noted 78% impairment using a telephone questionnaire (Woo et al., 2020). The small sample size and use of a questionnaire may have elevated the incidence in comparison with our study. A cohort study demonstrated cognitive decline in 50 mild COVID-19 cases compared to 28 non-infected controls at 6 months post-infection. However, comparison is difficult as they used a screening test and did not define cognitive impairment (Del Brutto et al., 2021). A case series study of seven mild-to-moderate cases indicated 100% impairment based on screening tests (Matos et al., 2021). Further research is needed to determine the incidence and characteristics of cognitive impairment in non-critical COVID-19.

Consistent with previous studies of critical cases, we demonstrated that executive function was the most affected cognitive domain (Helms et al., 2020; Zhou et al., 2020; Ferrucci et al., 2021; Hampshire et al., 2021; Jaywant et al., 2021; Mazza et al., 2021; Miskowiak et al., 2021; Raman et al., 2021). COVID-19 could disrupt executive attention network through several mechanisms including the olfactory neuronal pathway and neuroinflammation. Coronaviruses are neurotropic, traveling along axons (Bougakov et al., 2021). Given the high incidence of anosmia following COVID-19 infection, axon transport from the nasal cavity to the brain *via* the olfactory nerve is strongly suspected (Lu et al., 2020; Politi et al., 2020). Olfaction and executive function have common neurocircuitry in prefrontal and orbitofrontal cortices, and olfaction is considered a marker of cognitive function in neuropsychiatric and neurologic conditions (Fagundo et al., 2015; Zhou et al., 2019). Executive prefrontal cortex is a primary target of cytokine activation (Miller et al., 2013). Cytokines have been shown to decrease certain neurotransmitters, such as dopamine, which is pervasive in these regions (Miller et al., 2013). However, prefrontal networks also subserve other cognitive domains including attention, processing speed, and verbal memory, which were also associated with impairment.

The contribution of demographic, clinical, and psychosocial factors to cognitive impairment in COVID-19 survivors remains unclear. We found no sex differences in overall objective cognitive impairment, similar to two prior studies (Amalakanti et al., 2021; Hampshire et al., 2021). One prior study observed higher incidence of working memory impairment in female compared to male COVID-19 patients (Mazza et al., 2021). However, females also showed higher psychological distress, which may have exacerbated working memory difficulties. We observed greater frequency of impairment on a test of attention and processing speed in males. Previous reports of healthy adults suggested a female advantage for this test though these studies are quite outdated (Snow and Weinstock, 1990). COVID-19 research indicates that males have enhanced COVID-19 severity and mortality compared to females (Maleki Dana et al., 2020). This may reflect differences in the interactions among immune response and angiotensin-converting enzyme 2 (ACE2) expression related to X inactivation as well as the effects of sex hormones on these pathways (Viveiros et al., 2021). Our group and others have shown that males tend to have poorer cognitive outcomes following certain neurologic conditions, especially those involving X chromosome effects (Kesler et al., 2009a,b; Green et al., 2019). However, females also have increased susceptibility to certain brain-based disorders including depression and Alzheimer’s disease, for example (Kuehner, 2017; Duarte-Guterman et al., 2020). Our sample was largely female so other potential sex effects in cognitive and psychosocial outcomes may not have been detected.

Our findings suggest that a subgroup of younger individuals may be more vulnerable to cognitive and psychosocial effects of COVID-19. COVID-19 cases have increased in younger adults over time (Boehmer et al., 2020; Leidman et al., 2021) and even though COVID-19 tends to be less severe in younger adults, this cohort is still at much greater risk for COVID-19-related neurologic complications than from a typical influenza virus, for example (Fifi and Mocco, 2020). Young adulthood is a critical developmental stage when individuals tend to be actively engaging in higher education, establishing their careers, and becoming independent, making this a potentially vulnerable period.

We also found that younger participants had greater psychological distress. Consistent with these findings, other studies have noted higher incidence of pandemic-related psychological distress in younger adults (Varma et al., 2021). It has been suggested that younger adults may have less ability to regulate negative emotions surrounding the pandemic compared to older adults (Knepple Carney et al., 2021). Furthermore, some studies indicate that young adults may be at the highest risk for psychological sequelae related to loneliness and social isolation (Beam and Kim, 2020; Bu et al., 2020). Mazza and colleagues demonstrated that psychological symptoms were predictive of cognitive impairment in patients with COVID-19 (Mazza et al., 2021) but another study showed no correlation between these (Jaywant et al., 2021).

Our results demonstrated a correlation between psychological symptoms and subjective but not objective cognitive impairment. Psychological symptoms and subjective cognitive impairment were both self-reported and therefore influenced by the same response biases inherent in such measures. However, self-report measures allow for a more ecological assessment as they ask regarding symptoms in daily life across multiple days compared to objective measures which only measure function during the testing epoch. However, it is important to note that our sample was young and highly educated, and therefore, we were limited in ability to examine cognitive function in older and/or less educated individuals.

Younger age was significantly correlated with racial/ethnic minority status. Also, when examined together with age and psychological function, minority status showed the highest effect size, though was not statistically significant in our sample. Most studies conducted to date on COVID-related cognitive outcomes have not reported race/ethnicity data. Previous research has suggested that cognitive outcomes in other populations tend to be worse in minority individuals (Colby and Kraemer, 1975; Zhang et al., 2016). However, health disparities and the bias against minorities inherent in neuropsychological testing are often not addressed in such cognitive studies (Rivera Mindt et al., 2010; Cory, 2021). Future research should investigate the potential contribution of racial bias and discrimination on cognitive outcomes following COVID-19.

COVID-19 symptoms/severity were not associated with overall objective cognitive function but moderate severity disease was correlated with attention/processing speed impairment. We also noted that pandemic-related behavior, specifically, lower social distancing compliance, was associated with cognitive impairment. Males endorsed significantly lower social distancing compliance compared to females, but gender was not a significant mediator of the relationship between social distancing and cognitive impairment. Thus, gender and social distancing appear to contribute independently to cognitive impairment. As shown in Figure 1, the relationship between social distancing and cognitive function likely involves COVID symptoms and severity. It is also possible that lower cognitive function results in lower compliance with social distancing guidelines. Throughout the pandemic, younger adults have displayed less concern regarding the virus and lower adoption of social distancing measures compared to older adults (Atchison et al., 2020; Canning et al., 2020). However, we did not observe a relationship between age and social distancing.

The present study provides novel data regarding demographic factors related to cognitive impairment in persons with a history of mild/moderate COVID-19 infection but study limitations should be considered. The study design was cross-sectional; therefore, we could not determine if impairments were present pre-COVID or if cognitive trajectories change across time. Our study was consistent with prior reports in that we used test population norms for determining impairment. However, a well-matched control group would be more ideal as the study sample may differ demographically from the normative sample resulting in bias, especially for minorities. We used a common definition of cognitive impairment, but alternate definitions may yield different results. Our sample selection relied on self-reports, which may be influenced by recall biases. We were able to identify some individuals who likely incorrectly classified their disease severity, but it is possible that our sample may have included some participants who had more severe disease. Given the pandemic-related shutdown, this study was completed remotely which required computer and Internet access that may have limited and/or biased our sample. Although we conducted the testing under videoconferencing supervision, the results may have been negatively affected by the remote testing methodology. Our sample was comprised largely of young, highly educated individuals, reducing generalizability of the results. Cognitive effects of COVID-19 may vary geographically given the differences in regional pandemic response, attitudes, and policies, and therefore, larger, multisite studies are required.

Given the current decline in COVID-19 vaccination rates and the increase in cases due to viral variants, there will unfortunately remain a large population of survivors who are at risk for post-COVID syndrome. Continued research is required to address questions, such as which patients are at highest risk, what mechanisms underlie these symptoms and what interventions may be effective in reducing or reversing these symptoms.

## Data Availability Statement

The raw data supporting the conclusions of this article will be made available by the authors, without undue reservation.

## Ethics Statement

The studies involving human participants were reviewed and approved by University of Texas at Austin Institutional Review Board. The patients/participants provided their written informed consent to participate in this study.

## Author Contributions

SK and AH designed the project. AH, KL, and EG collected the data. SK analyzed the data and wrote the manuscript. All authors contributed to the article and approved the submitted version.

## Funding

This research was funded in part by the National Institutes of Health (R01CA226080 to SK, K01NR018970 to AH, and T32NR019035 to EG).

## Conflict of Interest

The authors declare that the research was conducted in the absence of any commercial or financial relationships that could be construed as a potential conflict of interest.

## Publisher’s Note

All claims expressed in this article are solely those of the authors and do not necessarily represent those of their affiliated organizations, or those of the publisher, the editors and the reviewers. Any product that may be evaluated in this article, or claim that may be made by its manufacturer, is not guaranteed or endorsed by the publisher.
